# Vertebral artery tortuosity in Turner syndrome: is tortuosity a component of the aortopathy phenotype?

**DOI:** 10.1186/1532-429X-16-S1-P115

**Published:** 2014-01-16

**Authors:** Shaine A Morris, William A Payne, Ronald V Lacro, Shiraz A Maskatia, Prakash Masand, Cory V Noel, Dianna M Milewicz, Rajesh Krishnamurthy

**Affiliations:** 1 Pediatric Cardiology, Texas Children's Hospital, Houston, Texas, USA; 2 Cardiovascular Clinical Research Core, Texas Children's Hospital, Houston, Texas, USA; 3 Cardiology, Boston Children's Hospital, Boston, Massachusetts, USA; 4 Pediatric Radiology, Texas Children's Hospital, Houston, Texas, USA; 5 Medical Genetics, University of Texas Medical School Houston, Houston, Texas, USA

## Background

Turner syndrome is associated with bicuspid aortic valve (BAV), coarctation of the aorta, aortic dilation, and aortic dissection. Vertebral artery tortuosity, as demonstrated by magnetic resonance angiography (MRA), is increased in other disorders associated with thoracic aortic dilation and dissection, including Marfan syndrome and Loeys-Dietz syndrome, and increased tortuosity is associated with earlier adverse cardiovascular outcomes in those groups. We investigated the association between vertebral artery tortuosity and aortic pathology in patients with Turner syndrome.

## Methods

We performed a retrospective analysis of 26 children and young adults with Turner syndrome who underwent cardiovascular MRA that included the neck in its coverage. Using a volume-rendering technique, we calculated the vertebral artery index (VTI), which is the maximum distance factor [(actual/straight length−1) × 100] of the two vertebral arteries. In patients with >1 MRA, the study with greater coverage of the vertebral arteries was used. Aortic root and ascending aortic dimensions were measured on MRA using oblique reformatting, and z-scores based on body surface area were calculated. Aortic z-score ≥2.0 was considered dilated. Associations between VTI, age, BAV, coarctation, aortic root (AR) z-score, and ascending aorta (AscAo) z-score were investigated in univariable and multivariable analyses.

## Results

Median age at MRA was 11.4 years (range 3.2-33.8). Seventeen patients (61%) had BAV, 9 (32%) had history of coarctation, and 8 (29%) had partial anomalous pulmonary venous return (PAPVR). None had aortic dissection. The mean AR z-score was 2.5 ± 1.9, and AscAo z-score 2.9 ± 3.1. Older age was associated with higher AR z-score (p = 0.028), while both older age and presence of BAV were associated with higher AscAo z-score (p = 0.010 and p = 0.013 respectively). VTI was significantly higher in patients with history of coarctation than without, with mean VTI 8.3 ± 4.5 vs. 4.9 ± 2.6, p = 0.021. Higher VTI was also associated with higher AR z-score (Pearson R = 0.433, p = 0.027) and higher AscAo z-score (R = 0.500, p = 0.009). VTI did not differ by BAV status or age. In multivariable analysis evaluating associations with aortic dilation, the associations between older age and VTI with AR z-score were not quite significant (p = 0.054 and 0.052 respectively). Older age (0.005), BAV (p = 0.005), and higher VTI (p = 0.026) remained independently associated with higher AscAo z-score. Patients post growth hormone (GH) had lower VTIs and poorer correlation between VTI and aortic dimensions than in patients not receiving GH.

## Conclusions

In Turner syndrome, increased vertebral artery tortuosity on CMRA is associated with coarctation of the aorta, increased AR z-score, and increased AscAo z-score, independent of BAV. This suggests that vertebral artery tortuosity may be a characteristic of the aortopathy in Turner syndrome. Further studied need to be done to determine if VTI is associated with cardiovascular outcomes in Turner syndrome.

## Funding

Baylor College of Medicine Cardiovascular Research Initiative Pilot Award.

**Figure 1:**
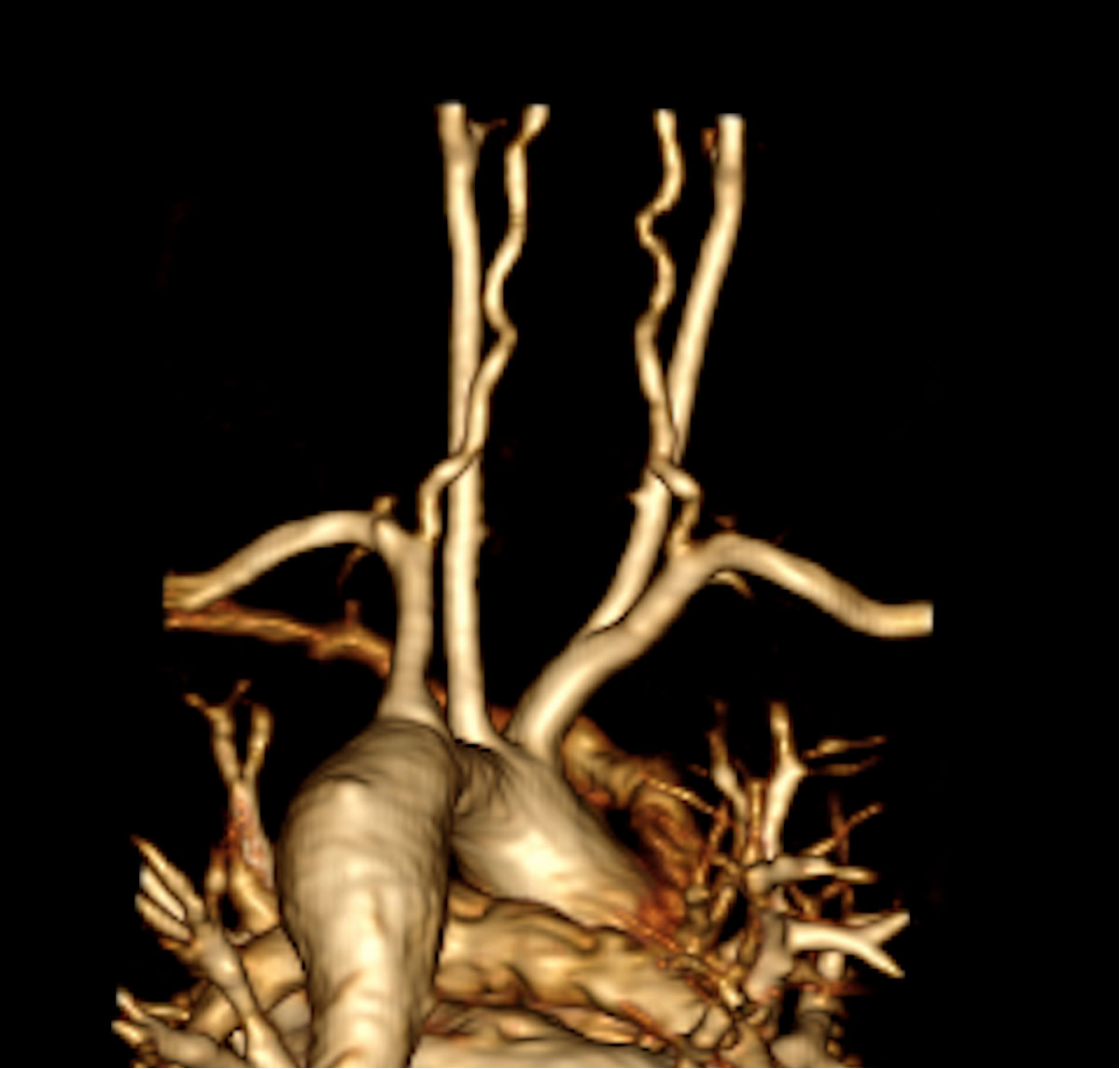
**Volume-rendered contrast-enhanced magnetic resonance angiogram of 18-year-old patient with Turner syndrome status post coarctation repair, with aortic dilation and vertebral artery tortuosity (VTI = 19)**.

